# Impact of Smoking and Chewing Tobacco on Arsenic-Induced Skin Lesions

**DOI:** 10.1289/ehp.0900728

**Published:** 2009-11-03

**Authors:** Anna-Lena Lindberg, Nazmul Sohel, Mahfuzar Rahman, Lars Åke Persson, Marie Vahter

**Affiliations:** 1 Institute of Environmental Medicine, Karolinska Institutet, Stockholm, Sweden; 2 URS Nordic AB, Stockholm, Sweden; 3 International Maternal and Child Health, Uppsala University, Uppsala, Sweden; 4 International Centre for Diarrhoeal Disease Research, Bangladesh, Dhaka, Bangladesh

**Keywords:** arsenic, interactions, metabolism, skin lesions, smoking, tobacco, urine metabolites

## Abstract

**Background:**

We recently reported that the main reason for the documented higher prevalence of arsenic-related skin lesions among men than among women is the result of less efficient arsenic metabolism.

**Objective:**

Because smoking has been associated with less efficient arsenic methylation, we aimed to elucidate interactions between tobacco use and arsenic metabolism for the risk of developing skin lesions.

**Methods:**

We used a population-based case–referent study that showed increased risk for skin lesions in relation to chronic arsenic exposure via drinking water in Bangladesh and randomly selected 526 of the referents (random sample of inhabitants > 4 years old; 47% male) and all 504 cases (54% male) with arsenic-related skin lesions to measure arsenic metabolites [methylarsonic acid (MA) and dimethylarsinic acid (DMA)] in urine using high-performance liquid chromatography (HPLC) and inductively coupled plasma mass spectrometry (ICPMS).

**Results:**

The odds ratio for skin lesions was almost three times higher in the highest tertile of urinary %MA than in the lowest tertile. Men who smoked cigarettes and *bidis* (locally produced cigarettes; 33% of referents, 58% of cases) had a significantly higher risk for skin lesions than did nonsmoking men; this association decreased slightly after accounting for arsenic metabolism. Only two women smoked, but women who chewed tobacco (21% of referents, 43% of cases) had a considerably higher risk of skin lesions than did women who did not use tobacco. The odds ratio (OR) for women who chewed tobacco and who had ≤ 7.9 %MA was 3.8 [95% confidence interval (CI), 1.4–10] compared with women in the same MA tertile who did not use tobacco. In the highest tertile of %MA or %inorganic arsenic (iAs), women who chewed tobacco had ORs of 7.3 and 7.5, respectively, compared with women in the lowest tertiles who did not use tobacco.

**Conclusion:**

The increased risk of arsenic-related skin lesions in male smokers compared with nonsmokers appears to be partly explained by impaired arsenic methylation, while there seemed to be an excess risk due to interaction between chewing tobacco and arsenic metabolism in women.

Even moderately elevated concentrations of inorganic arsenic (iAs) in drinking water are of major public health concern worldwide [World Health Organization (WHO) 2001]. In Bangladesh alone, more than 50 million inhabitants are drinking water that contains arsenic above the WHO guideline value of 10 μg/L [British Geological Survey (BGS) 2001; Chakraborti et al. 2004]. In addition, there is increasing concern about arsenic exposure from food, especially rice products (Sun et al. 2008). Chronic arsenic exposure is associated with an increased risk of cancer of the skin, lungs, bladder, liver, and possibly kidneys [International Agency for Research on Cancer (IARC) 2004a], as well as a number of noncarcinogenic effects (Rahman et al. 2007; States et al. 2009; WHO 2001). The earliest signs of toxicity from chronic exposure to iAs in humans are pigmentation changes and hyperkeratosis, which may proceed to skin cancer (Yu et al. 2006). Unlike the arsenic-related cancers, which may appear first after two to three decades of exposure, these skin lesions may appear within a few years of exposure (Chen et al. 2006; Saha 2003).

A wide variation appears to exist in susceptibility to arsenic-induced toxicity. The best documented risk-modifying factor for arsenic-related health effects is the metabolism of arsenic. iAs is methylated via one-carbon metabolism using arsenic (III) methyltransferase (AS3MT). The main metabolites excreted in urine are methylarsonic acid (MA) and dimethylarsinic acid (DMA), besides some unmethylated iAs, but major differences exist between individuals and population groups (Vahter 2002). Numerous studies have demonstrated positive associations between the percentage of MA in urine and various cancers, chromosome aberrations, and oxidative stress (Chen et al. 2003a, 2003b, 2005a, 2005b; Chung et al. 2008; Hsueh et al. 1997; Huang et al. 2008; Li et al. 2008; Mäki-Paakkanen et al. 1998; Pu et al. 2007; Steinmaus et al. 2006; Xu et al. 2008; Yu et al. 2000). Two recent studies (Ahsan et al. 2007; Lindberg et al. 2008b) also reported increased risk for pigmentation changes and hyperkeratosis.

Another known risk-modifying factor is cigarette smoking. For quite some time, researchers have known that concurrent exposure to arsenic and smoking synergistically increases the risk of lung cancer (Mostafa et al. 2008; Pershagen et al. 1987), and experimental studies showed that arsenic and cigarette smoke at environmentally relevant levels act synergistically to cause DNA damage (Hays et al. 2006). Similarly, bladder cancer seemed to be significantly associated with arsenic exposure among smokers only (Bates et al. 2004; Steinmaus et al. 2003). Recently, an interaction between elevated arsenic exposure via drinking water and tobacco smoking was indicated also for the induction of skin lesions (Chen et al. 2006).

It was recently reported that the well- documented less efficient methylation of arsenic in men, compared with women (Vahter et al. 2007), is the main reason for the observed higher prevalence of skin lesions in men (Lindberg et al. 2008b). Because smoking has been associated with less efficient arsenic methylation (Hopenhayn-Rich et al. 1996; Lindberg et al. 2008a), we evaluated potential interactions between smoking and arsenic metabolism for the risk of developing skin lesions.

## Materials and Methods

### Study population

This study is part of a population-based case–referent study concerning the development of skin lesions in relation to arsenic exposure via drinking water carried out in Matlab, a rural area 53 km southeast of Dhaka, Bangladesh (Rahman et al. 2006a, 2006b). More than 60% of the tube wells in this area have concentrations above 50 μg/L, which is the Bangladeshi standard for drinking water, and 70% are above the WHO maximum guideline of 10 μg As/L. In Matlab, the International Centre for Diarrhoeal Disease Research, Bangladesh (ICDDR,B) has been running a comprehensive Health and Demographic Surveillance System (HDSS) that covers a population of 220,000.

We obtained informed consent from all participants, and the study was approved by both the ICDDR,B Ethical Review Committee and the ethics committee at the Karolinska Institutet in Stockholm. Mitigating activities such as painting wells with elevated arsenic concentrations and installing filters were initiated as described elsewhere (Jakariya et al. 2005; Rahman et al. 2006b).

### Cases and referents recruitment

The recruitment of persons with skin lesions (504 cases) and the selection of referents (1,830 referents) are described elsewhere (Rahman et al. 2006a). In short, all residents > 4 years of age who had lived in the study area for at least 6 months were eligible for the present study (180,811 individuals). In total, 166,934 (92%) of the eligible individuals were interviewed and examined for skin lesions by well-trained field teams, and 1,682 suspected cases were referred to the study physicians (one male and one female) at the health centers. Eventually, 504 cases were diagnosed with arsenic-induced skin lesions (defined as hyperpigmentation, hypopigmentation, and keratosis). For details regarding the ascertainment of cases, see Rahman et al. (2006a). All cases provided urine samples. Referents were randomly selected from the HDSS database with the criteria of more than 4 years of age, living in the area for at least 6 months, and drinking water from the area at least once a week. Selected referents were interviewed and invited to attend the clinic to be examined for skin lesions by a physician and to provide urine samples. A total of 1,579 referents attended the clinic.

### Data collection

The field teams interviewed all individuals about their history of water consumption and the water sources used, including location, during each calendar year since 1970 or since birth if later than 1970, as described previously in more detail (Rahman et al. 2006b). Data on socioeconomic status (SES) were collected from the HDSS database and were defined in terms of assets relevant to these rural settings (Rahman et al. 2006b). Information on tobacco use, obtained in the interviews, was divided into cigarette smoking, *bidi* (locally produced cigarettes) smoking, or chewing tobacco, the latter consisting of dried tobacco leaves or zarda (a type of chewing tobacco, often used with areca nut, slaked lime, and betel leaves). Water samples were collected from all functional wells in the area and stored at −20°C (*n* = 13,286). Arsenic concentrations in drinking water were determined using atomic absorption spectrometry with hydride generation, with addition of hydrochloric acid and potassium iodine combined with heating (Wahed et al. 2006). For samples with concentrations below the limit of detection (LOD; 1.0 μg/L), half the LOD was used in the calculations.

### Arsenic exposure

Both the average and the cumulative historical arsenic exposure were calculated as the time-weighted mean arsenic concentration of drinking water of all sources used since 1970 or birth. The cumulative arsenic exposure was calculated by summing the arsenic concentration multiplied by the number of years of usage (micrograms per liter × years) for all water sources used since 1970. From the interviews of the participants regarding their water consumption history, we were able to collect data on which year the participant started to drink well water (after 1970) and thereby the age at first exposure to tube well water. Very few wells were constructed before 1970, at which time the registration of the wells in the HDSS began.

### Urine arsenic measures

Spot urine samples were collected in 20 mL polyethylene containers and stored at −20°C. We randomly selected 526 samples from all referents and all 504 cases for analysis of arsenic metabolites in urine for evaluation of the individual arsenic methylation efficiency. The individual pattern of arsenic metabolites in urine is shown to be fairly stable over time (Concha et al. 2002; Kile et al. 2009; Steinmaus et al. 2005). Speciation analysis of arsenic metabolites in urine was performed by an inductively coupled plasma mass spectrometer (ICP-MS; Agilent 7500ce, Agilent Technologies, Waldbronn, Germany) together with an Agilent 1100 chromatographic system equipped with solvent degasser, auto sampler, and temperature-controlled column. For the separation of trivalent arsenic [As(III)], MA, DMA, and pentavalent arsenic [As(V)], a Hamilton PRP-X100 anion exchange column (4.6 mm × 250 mm) was used (Lindberg et al. 2006, 2007). For quality control, we used a Japanese reference urine certified for arsenic (Yoshinaga et al. 2000) and a spiked urine sample. Mean concentrations of As(III), DMA, MA, and As(V) in the reference urine were 3.9 ± 0.4, 43 ± 3, 3.0 ± 0.4, and 0.1 ± 0.1 (*n =* 79 during an 8-week period), respectively. The mean concentrations of the spiked urine samples of As(III), DMA, MA, and As(V) were 1.5 ± 0.6, 58 ± 3, 10 ± 0.6, and 16 ± 1.3 (*n =* 82 during an 8-week period), respectively. The results of interlaboratory comparisons are described in more detail elsewhere (Lindberg et al. 2007). Arsenic concentrations in urine were adjusted to the average specific gravity in this population (1.012 g/cm^3^), as measured by a refractometer (Uricon-Ne, ATAGO Co. Ltd, Tokyo, Japan) to adjust for variation in dilution in the urine samples (Nermell et al. 2008).

We also measured the arsenic concentrations of various forms of cigarettes, using ICP-MS after microwave-assisted acid digestion at high temperature and pressure as previously described for blood and breast milk (Fangstrom et al. 2008).

### Statistical analyses

We used SPSS 14.0 for Windows (SPSS Inc., Chicago, IL, USA) to perform all statistical analyses. Multivariate logistic regression analyses were performed to estimate the odds ratios (ORs) and corresponding confidence intervals (CIs) for having skin lesions at different proportions of arsenic metabolites in urine. Continuous variables were stratified into tertiles when estimating the ORs for having skin lesions. All multivariate associations were simultaneously adjusted for sex (man/woman), age (continuous), SES (continuous), tobacco use (no/yes), and cumulative arsenic exposure (continuous). We chose to adjust all models for cumulative arsenic exposure instead of average arsenic exposure or urinary arsenic concentrations. The cumulative arsenic exposure influenced the associations slightly more than did the average arsenic exposure, and adjusting for the urinary arsenic concentrations did not make any difference in the models when tested (data not shown). We calucated *p* for trend by treating the categorical variables as continuous variables in the models. To evaluate potential biologic interactions between the different risk factors, that is, arsenic metabolism and smoking, on arsenic-related skin lesions, we calculated the relative excess risk due to additive interactions (RERI) and corresponding CIs (Ahlbom and Alfredsson 2005). These calculations were performed according to Andersson et al. (2005), using an Excel sheet (EpiNET 2008). We used *p*-values < 0.05 for statistical significance.

## Results

Sex-specific characteristics of the cases and referents are presented in Table 1. The cases were more often men than women (54% vs. 46%) and older than the referents (overall, 40 vs. 31 years old). The cases also had higher SES, as measured by household asset scores, and they smoked cigarettes or *bidis* (men only) or used zarda more often than did the referents. The three different measures of arsenic exposure, that is, cumulative lifetime exposure to arsenic in drinking water (micrograms per liter × years), average lifetime exposure to arsenic (micrograms per liter), and current total exposure to iAs, as measured by the concentration of arsenic metabolites in urine, are also presented in Table 1. The cases had significantly higher lifetime cumulative arsenic exposure and average lifetime arsenic exposure than did the referents but not higher urinary arsenic concentrations.

The number of years of tobacco use and the number of tobacco products used per day among cases and referents are shown in Supplemental Material, Table S1 (doi:10.1289/ehp.0900728). The pattern of tobacco use differed markedly between men (46% smokers, 11% chewing tobacco) and women [only two female smokers, 31% chewing tobacco (see Supplemental Material, Table S2)]. We analyzed men and women separately. The associations between the different forms of tobacco use and urinary arsenic metabolites are shown in Table 2. All forms of tobacco use were associated with an increased percentage of MA and a decreased percentage of DMA, whereas the percentage of iAs and the urinary arsenic concentration did not vary much by tobacco use.

Men who smoked cigarettes or *bidis* had significantly higher risk for skin lesions than did men who did not use tobacco (OR = 1.8; 95% CI, 1.1–3.1; adjusted for age, SES, and cumulative arsenic exposure; Table 3). We observed a marked drop in the OR after adjusting for age, SES, and cumulative arsenic exposure (crude OR = 3.2). Entering the different covariables independently showed that age was the main confounding factor. The adjusted OR in men chewing tobacco was not significantly increased (adjusted OR = 0.80; 95% CI, 0.36–1.7; Table 3); however, the number of individuals was low. Because very few women smoked, we could not determine the effect on the risk for skin lesions among women. The multivariable-adjusted model that included all women who used tobacco (mainly chewing tobacco) showed that they had considerably higher prevalence of skin lesions than did women who did not use tobacco (adjusted OR = 2.4; 95% CI, 1.4–3.9; Table 3). Excluding the two women who smoked cigarettes did not change the associations. To test for the influence of arsenic methylation on the association between tobacco use and arsenic-related skin lesions, we also adjusted the OR values for %MA in urine. As shown in Table 3, the OR for men who smoked changed from 1.8 to 1.4 (95% CI, 0.80–2.4) after additional adjustment for %MA. Compared with women who did not use any tobacco, the OR for women who chewed tobacco changed from 2.4 to 2.0 (95% CI, 1.2–3.4) after adjusting for %MA and remained significantly increased.

To further evaluate interactions between smoking and arsenic metabolism on the risk for skin lesions, we stratified both tobacco use and urinary arsenic metabolites and analyzed the joint effects. The increasing risk for skin lesions with increasing proportion of MA and decreasing proportion of DMA was more pronounced among nontobacco using men and women (Table 4). The OR for men who used tobacco (mostly smokers, because few chewed tobacco) with ≤ 7.9 %MA was 1.8 (95% CI, 0.53–6.3; *p* = 0.34), compared with men in the same tertile of %MA who did not use tobacco. Men within the highest tertile of %MA who used tobacco had an OR of 4.4 (95% CI, 1.7–11). This OR was essentially the same as the sum of the OR for nontobacco users in the highest %MA tertile and that for tobacco users in the lowest %MA tertile minus the baseline OR (OR values 3.8 + 1.8 − 1.0 = 4.6). Similar results were obtained for %iAs; the OR_joint_ was 2.8, which is approximately equal to the sum (minus 1) of the OR for nonsmokers in the highest %iAs tertile (OR 1.6) and that for smokers in the lowest %iAs (OR = 1.9). Among women, the OR for tobacco users (mainly chewing tobacco) with ≤ 7.9 %MA in urine was 3.8 (95% CI, 1.4–10), compared with nonusers of tobacco within the same tertile (*p* = 0.009; Table 5). Women in the highest %MA tertile who chewed tobacco had an OR of 7.3 (95% CI, 3.4–15), compared with women in the lowest %MA group who did not use tobacco. This OR was higher than the predicted additive risks (5.7), although the RERI was not statistically significant (1.5; 95% CI, −3.7 to 6.7). Similarly, the OR_joint_ for women in the highest %iAs tertile who used tobacco was 7.5, which was higher than the predicted additive risks (2.5). The RERI was 5.0 (95% CI, −1.3 to 11.4), the attributable proportion due to interaction was 0.67 (0.36–0.98), and the synergy index was 4.4 (1.2–16), which indicated a biologic interaction.

## Discussion

This population-based case–referent study in Bangladesh is the first to evaluate the combined effects of arsenic exposure, arsenic metabolism, and use of tobacco for the risk of arsenic-related skin effects. All forms of tobacco use were associated with less efficient methylation of arsenic. Among men, there appeared to be an additive effect of poor arsenic methylation (high iAs and high %MA) and smoking for the development of arsenic-induced skin lesions, although a high %MA increased the risk more than did smoking. Because very few women smoked cigarettes or *bidis*, an interaction between arsenic methylation and smoking in women could not be evaluated. Another new finding in the present study was that tobacco chewing, which is much more common among Bangladeshi women than smoking, was also a risk factor for developing arsenic-related skin lesions in women. The high ORs for skin lesions among the women who chewed tobacco in the highest tertiles of %iAs or %MA (7.5 and 7.3, respectively), compared with nontobacco using women with efficient arsenic methylation, suggest an interaction, although the RERI values were not quite significant. For men, an association between chewing tobacco and skin lesions was observed in the crude analysis only, but the sample size was small and the CIs wide. Further studies on larger cohorts are warranted for firm conclusions concerning the biologic interactions between various tobacco use, arsenic exposure, and arsenic metabolism. In any case, the use of various forms of tobacco should be considered in the risk assessment of arsenic and in the comparison of arsenic-related health risks among populations.

Tobacco smoking has been identified as an independent risk factor of nonmelanoma skin cancer (De Hertog et al. 2001; Grant 2008; Grodstein et al. 1995), psoriasis (Setty et al. 2007), and premature skin aging (Just et al. 2007; Morita 2007), but few studies have investigated the modifying effect of smoking on the arsenic-related hyperpigmentation and hyperkeratosis. Chen et al. (2006) reported a significant synergistic effect between the highest level of arsenic exposure via drinking water (> 113 μg/L) and tobacco smoking for the risk of skin lesions among Bangladeshi men, but much weaker interaction effects among women. These authors suggested that the interaction could be due to immunosuppression caused by tobacco smoking, inhibition of arsenic methylation, or the prevalent smoking of *bidis*, the filterless, locally produced cigarettes with raw tobacco. *Bidis* are popular in rural areas in Bangladesh and are claimed to contain more carcinogenic substances than do cigarettes.

In the present study, the effect of smoking on arsenic-related skin lesions was studied only in men, because, by tradition, very few women in Bangladesh are smokers. According to personal interviews, almost half the men were smokers, whereas only two of more than 500 women smoked. It is highly unlikely that we have any differential misclassification in the data on tobacco use. Smoking or other forms of tobacco use are not linked to any stigma and are in no way considered to be associated with the arsenic-induced skin lesions by the study population. The increased risk of skin lesions among men who smoked was not due to additional exposure to arsenic via smoking. The concentrations of arsenic in different brands of cigarettes from local shops in Matlab ranged between 0.13 and 0.29 μg/g (mean 0.21 μg/g, *n* = 5) and between 0.24 and 0.27 μg/g (mean 0.25 μg/g, *n* = 3) for *bidis*. Thus, it could be estimated that the inhaled amount of arsenic by smoking 10 cigarettes or *bidis*/day, for example, was about 2 μg. Even though a considerable part of this arsenic is absorbed in the lungs, the arsenic uptake from smoking is negligible compared with that from drinking water (70% of the wells had > 10 μg As/L; Rahman et al. 2006b) and food (Lindberg et al. 2008a).

Instead, we found that smoking was associated with higher %MA in urine, which is a known risk factor for arsenic-related skin effects (Ahsan et al. 2007; Chen et al. 2003a; Del Razo et al. 1997; Hsueh et al. 1997; Lindberg et al. 2008a; Yu et al. 2000). Elevated %MA in urine may be related to the highly toxic trivalent intermediate metabolite MA(III) (Ganyc et al. 2007; Vega et al. 2001) in the tissues, including skin (Vahter 2002). The decrease in the second step in the methylation of arsenic (to DMA) by smoking is in agreement with previous findings (Hopenhayn-Rich et al. 1996; Lindberg et al. 2008b); however, the mechanism by which this occurs is not clear. It may be that smoking inhibits the specific AS3MT involved in arsenic methylation or impairs one-carbon metabolism in general. Cigarette smoking is known to increase serum homocysteine concentration (O’Callaghan et al. 2002; Refsum et al. 2006), which, via the concurrent accumulation of *S*-adenosylhomocysteine, exerts a strong inhibition of *S*-adenosylmethionine–dependent transmethylation reactions, including those of arsenic (Gamble et al. 2005; Marafante and Vahter 1984). Smokers also tend to have lower levels of folate and vitamin B6 and B12 (O’Callaghan et al. 2002), all of which are essential for homocysteine metabolism. The smoking-related increase in homocysteine is most likely less pronounced in women, in whom the estrogen-dependent upregulation of endogenous choline synthesis may, via oxidation to betaine, contribute to remethylation of homocysteine (Zeisel 2007). Indeed, the study by Chen et al. (2006) observed a much stronger effect of smoking on arsenic-related skin lesions in men than in women.

Another new finding in this study is that chewing tobacco is a risk factor for arsenic-related skin effects for women and that there appears to be an interaction with poor metabolism of arsenic. For men, the effect of chewing tobacco on the risk of skin lesion seems to be much less, but the number of cases is too small for a firm conclusion. About 31% of the women reported chewing tobacco, which locally is called shada (dried tobacco leaves) or zarda (processed tobacco leaves in a paste) (Choudhury et al. 2007). In zarda, the tobacco is often mixed with sliced areca nut, lime, and sometimes a leaf of the piper betel plant, and the adverse effects may be caused by this mixture rather than the tobacco alone. Areca nut, the seed of *Areca catechu*, is used in a variety of chewed products, often mixed with tobacco or betel leaves. Both betel quid and areca nut have been associated with increased risk for oral epithelial malignancy (IARC 2004b; Lee et al. 2008). In a previous study in Bangladesh, McCarty et al. (2006) reported that betel nut use, but not tobacco chewing or cigarette smoking, was associated with skin lesions. As that study matched by sex, it is not known if the associations were sex dependent. In the present study, zarda contained about half a microgram of arsenic per gram of tobacco (0.33–0.54 μg/g; mean 0.45 μg/g; *n* = 8), which is about twice as much as in cigarettes and *bidis*. Still, the arsenic exposure from chewing zarda was low compared with that from the drinking water and food.

## Figures and Tables

**Table 1:** ORs (95% CIs) for skin lesions by characteristics of case–referent participants (504 cases, 528 referents).

	Male			Female		
Group^a^	Referents (*n*)	Cases (*n*)	OR_crude_ (95% CI)	Referents (*n*)	Cases (*n*)	OR_crude_ (95% CI)
Age (years)						
≤ 18	93	3	1.0	85	8	1.0
18–39	68	116	53 (16–173)**	105	110	11 (5–24)**
> 39	88	153	54 (17–175)**	89	114	14 (6–30)**

SES^b^						
≤ −0.60	80	50	1.0	93	42	1.0
−0.60 to 0.74	81	77	1.5 (0.95–2.4)	91	81	2.0 (1.2–3.2)**
> 0.74	76	136	2.9 (1.8–4.5)**	86	99	2.5 (1.6–4.1)**

Tobacco use						
None	142	83	1.0	220	132	1.0
Cigarettes	39	93	4.1 (2.6–6.5)**	1	0	—
* Bidi*	44	64	2.5 (1.6–4.0)**	1	0	—
Chewing tobacco	24	32	2.3 (1.3–4.1)**	57	100	2.9 (2.0–4.3)**

CumAs^c^						
≤ 1,639	86	51	1.0	90	29	1.0
1,639–4,107	81	45	0.94 (0.57–1.5)	95	55	1.8 (1.1–3.1)*
> 4107	82	176	3.6 (2.3–5.6)**	94	148	4.9 (3.0–8.0)**

AverageAs^d^						
< 80	84	67	1.0	94	43	1.0
80–181	80	54	0.85 (0.53–1.4)	92	74	1.8 (1.1–2.8)*
> 181	85	151	2.2 (1.5–3.4)**	93	115	2.7 (1.7–4.3)**

U-As^e^						
≤ 51	78	87	1.0	96	80	1.0
51–124	90	75	0.75 (0.49–1.2)	89	56	0.76 (0.48–1.2)
> 124	81	110	1.2 (0.80–1.9)	94	96	1.2 (0.81–1.8)

a Tertiles of referents.

b Values refer to household assets.

c Cumulative arsenic exposure (μg/L × years).

d Average lifetime arsenic exposure (μg/L).

e Sum of arsenic metabolites in urine (μg/L).

* *p*< 0.05.

** *p* < 0.01.

**Table 2:** Pattern of arsenic metabolites (percentages of iAs, MA, DMA) in urine, by tobacco use and sex.

Group	*n*	U-As (μg/L) Median	%iAs		%MA		%DMA	
			Mean ± SD	Median	Mean ± SD	Median	Mean ± SD	Median
Men								
None^a^	225	81.2	13.1 ± 7.0	11.4	11.8 ± 5.5	10.9	75.1 ± 10.1	77.1
Cigarettes	132	97.8	14.2 ± 7.4	12.9	16.2 ± 5.6**	16.0	69.6 ± 10.9**	71.5
* Bidi*	108	86.6	15.2 ± 9.2*	12.2	15.5 ± 6.4**	14.5	69.3 ± 12.8**	71.8
Chewing tobacco	56	82.0	14.2 ± 7.0	13.3	15.8 ± 6.4**	14.7	70.0 ± 11.4**	71.9

Women								
None^a^	352	69.0	12.9 ± 7.5	11.2	10.1 ± 4.9	9.3	77.0 ± 9.9	79.3
Cigarettes	1	—	9.1	—	10.9	—	80.0	—
* Bidi*	1	—	8.9	—	13.0	—	78.1	—
Chewing tobacco	157	89.4	12.4 ± 8.0	10.5	12.7 ± 5.5**	12.2	74.9 ± 11.3*	77.4

a Reference group.

* *p* < 0.05.

** *p* < 0.01.

**Table 3:** ORs for arsenic-related skin lesions in relation to tobacco use among the 528 referents and 504 skin lesion cases, stratified by sex.

	Referents (*n*)	Cases (*n*)	OR_crude_ (95% CI)	OR_adj_ (95% CI)^a^	OR_methylation_ (95% CI)^b^
Men					
None	142	83	1.0	1.0	1.0
Cigarettes/*bidi*	83	157	3.2 (2.2–4.7)**	1.8 (1.1–3.1)*	1.4 (0.80–2.4)
Chewing tobacco	24	32	2.3 (1.3–4.1)**	0.80 (0.36–1.7)	0.60 (0.27–1.4)
Tobacco use	107	189	3.0 (2.1–4.3)**	1.6 (1.0–2.7)	1.2 (0.72–2.1)

Women					
None	220	132	1.0	1.0	1.0
Tobacco use^c^	59	100	2.8 (1.9–4.2)**	2.4 (1.4–3.9)**	2.0 (1.2–3.4)**

a Adjusted for age, SES, and cumulative arsenic exposure;

b Adjusted for age, SES, cumulative arsenic exposure, and %MA;

c Mainly tobacco chewing; only two women smoked.

* *p* < 0.05.

** *p* < 0.01.

**Table 4:** Joint effect of arsenic metabolites in urine and tobacco use for the risk of arsenic-related skin lesions among men.

Groups^a^	Referents (*n*)	Cases (*n*)	OR_strat_ (95% CI)^b^,^c^	*p*_trend_	OR_joint_ (95% CI)^c^
%iAs					
None					
≤ 9.5	49	22	1.0		1.0
9.5–13	42	25	1.3 (0.54–3.3)		1.2 (0.53–2.7)
> 13	51	36	2.2 (0.93–5.3)	0.07	1.6 (0.77–3.5)

Tobacco user^d^					
≤ 9.5	24	46	1.0		1.9 (0.8–4.4)
9.5–13	39	48	0.64 (0.32–1.3)		1.3 (0.58–2.9)
> 13	44	95	1.1 (0.57–2.3)	0.5	2.8 (1.3–5.8)**

%MA					
None					
≤ 7.9	48	8	1.0		1.0
7.9–12	51	21	1.1 (0.36–3.1)		1.3 (0.48–3.5)
> 12	43	54	3.3 (1.3–9.2)*	0.003	3.8 (1.5–9.6)**

Tobacco user^d^					
≤ 7.9	14	11	1.0		1.8 (0.53–6.3)
7.9–12	24	27	1.2 (0.42–3.3)		2.4 (0.80–6.9)
> 12	69	151	2.1 (0.86–5.2)	0.03	4.4 (1.7–11)**

%DMA					
None					
≤ 76	53	50	1.0		1.0
76–82	44	24	0.62 (0.28–1.4)		0.60 (0.29–1.2)
> 82	45	9	0.25 (0.090–0.71)**	0.008	0.31 (0.13–0.77)*

Tobacco user^d^					
≤ 76	65	140	1.0		1.4 (0.77–2.6)
76–82	29	32	0.47 (0.25–0.90)*		0.58 (0.27–1.3)
> 82	13	17	0.72 (0.30–1.7)	0.1	0.78 (0.29–2.1)

a Tertiles of referents.

b OR for the groups stratified by level of arsenic metabolites.

c Adjusted for age, SES, and cumulative arsenic exposure.

d Of the participants who used tobacco, 78% of the referents and 83% of the cases smoked cigarettes or *bidis*. See also Supplemental Material, Tables S1 and S2 (doi:10.1289/ehp.0900728).

* *p* < 0.05.

** *p* < 0.01.

**Table 5:** Joint effect of arsenic metabolites in urine and tobacco use for the risk of arsenic-related skin lesions among women.

Groups^a^	Referents (*n*)	Cases (*n*)	OR_strat_ (95% CI)^b^,^c^	*p*_trend_	OR_joint_ (95% CI)^c^
%iAs					
None					
≤ 9.5	74	40	1.0		1.0
9.5–13	82	32	0.96 (0.50–1.8)		0.89 (0.48–1.7)
> 13	64	60	1.9 (1.0–3.4)*	0.04	1.7 (0.95–3.1)

Tobacco use^d^					
≤ 9.5	28	33	1.0		1.8 (0.83–3.8)
9.5–13	23	27	0.85 (0.36–2.0)		1.9 (0.89–4.2)
> 13	8	40	2.5 (0.88–6.9)	0.1	7.5 (3.0–19)**

%MA					
None					
≤ 7.9	102	34	1.0		1.0
7.9–12	76	40	1.4 (0.74–2.5)		1.4 (0.76–2.5)
> 12	42	58	2.8 (1.5–5.2)**	0.002	2.9 (1.6–5.5)**

Tobacco use^d^					
≤ 7.9	13	16	1.0		3.8 (1.4–10)**
7.9–12	27	22	0.38 (0.12–1.2)		1.6 (0.73–3.6)
> 12	19	62	1.6 (0.55–4.8)	0.06	7.3 (3.4–15)**

%DMA					
None					
≤ 76	57	66	1.0		1.0
76–82	82	36	0.35 (0.19–0.64)**		0.37 (0.20–0.66)**
> 82	81	30	0.39 (0.21–0.72)**	0.002	0.39 (0.21–0.72)**

Tobacco use^d^					
≤ 76	12	51	1.0		3.2 (1.4–7.1)**
76–82	27	26	0.26 (0.10–0.66)**		0.66 (0.31–1.4)
> 82	20	23	0.40 (0.15–1.1)	0.06	0.98 (0.43–2.2)

a Tertiles of referents.

b OR for the groups stratified by level of arsenic metabolites.

c Adjusted for age, SES, and cumulative arsenic exposure.

d Mainly tobacco chewing; only two women smoked.

* *p* < 0.05.

** *p* < 0.01.
